# Rapid laboratory identification of fibrinogen Longmont: a case report and literature review

**DOI:** 10.3389/fmed.2026.1781076

**Published:** 2026-06-22

**Authors:** Cuihua Ma, Xuezhuo Li, Da Yin, Huidong Ma, Wenran Lu, Chunhua Wang

**Affiliations:** Department of Clinical Laboratory, First Hospital of Qinhuangdao, Qinhuangdao, China

**Keywords:** coagulation curve, dysfibrinogenaemia, fibrinogen, fibrinogen Longmont, methodological difference

## Abstract

This study reports a case of a patient with unstable angina with abnormally low fibrinogen (FIB) levels detected during preoperative screening, yet whose clinical manifestations were markedly inconsistent with the test results. Through comparative analysis of multiple methodologies, coagulation curve analysis, manual control, and normal plasma mixing tests, it was found that the optical FIB assay yielded falsely low or failed results (<0.35 g/L), while the magnetic bead assay gave normal results (4.18 g/L; reference range 2–4 g/L) in this case, demonstrating a marked methodological discrepancy. However, as demonstrated in the literature, FIB Longmont may cause method-, reagent-, platform-, and algorithm-dependent discrepancies, with optical signal loss being a major but not exclusive mechanism combined with genetic testing, the diagnosis was ultimately confirmed as a rare dysfibrinogenemia—fibrinogen Longmont. This study aims to explore the laboratory diagnostic characteristics and differential diagnostic approaches for this condition, providing a reference for accurate interpretation of coagulation reports and avoiding misdiagnosis and mistreatment in clinical practice.

## 1 Introduction

Fibrinogen (FIB) is a 340 kDa glycoprotein synthesized by the liver, composed of three pairs of polypeptide chains: Aα, Bβ, and γ. It serves as the substrate for thrombin-mediated fibrin clot formation. Over 400 congenital FIB gene variants have been reported to date, among which fibrinogen Longmont (FG-LM) is characterized by the substitution of arginine at position 196 with cysteine in the Bβ chain (p.Arg196Cys). The introduced free thiol group impairs lateral aggregation of protofibrils, resulting in a “transparent clot” with significantly increased translucency and reduced mechanical strength (1–3). Optical methods (based on turbidity changes) often report pseudo-low or failed FIB results due to reduced turbidity signal, whereas magnetic bead assays (based on viscoelasticity) are less affected by clot translucency and may yield normal results in many cases (1). However, the situation is more complex: Jennings et al. (4) demonstrated substantial inter-laboratory and inter-platform variability, and Leung et al. (3) showed that normal Clauss FIB results may occasionally be obtained on optical analyzers depending on the thrombin reagent used. Therefore, FG-LM can cause method-, reagent-, platform-, and algorithm-dependent discrepancies, with optical signal loss being a major but not exclusive mechanism. Additional observations include the absence of a clotting curve plateau, ΔmAbs (change in milli-absorbance units) below the instrument threshold, manually observed loose and transparent clots, and the mixed inhibitory effect of patient plasma on normal FIB. These laboratory findings provide a simple and rapid laboratory pathway for clinical identification of FG-LM, avoiding unnecessary blood product transfusions and surgical delays.

## 2 Case presentation

A 71-year-old man, was transferred from another hospital due to “paroxysmal chest tightness and pain for 2.5 months, with a recurrence 10 days ago.” The admission diagnosis was coronary atherosclerotic heart disease and unstable angina pectoris, with planned coronary percutaneous coronary intervention (PCI). Preoperative coagulation screening (ACL TOP-750, optical method, Instrumentation Laboratory Company, USA) showed: FIB-Clauss method <0.35 g/L (instrument linear lower limit), FIB-PT derivative method 0.93 g/L (reference range 2.76–4.71 g/L); PT and APTT were normal, TT18.9 s (10.3–16.6 s); AT75% (83–128%); D-D, FDP, and liver and kidney function were all normal. The following reagents were used: FIB (Clauss), PT, APTT, TT, D-dimer (immunoturbidimetry), FDP (immunoturbidimetry), and antithrombin (chromogenic substrate) kits, as well as coagulation assay diluent, all manufactured by Instrumentation Laboratory Company (USA). The patient’s medical history revealed no prior diagnoses of hypertension, diabetes mellitus, cerebral infarction, or cerebral hemorrhage. He had no history of hepatitis, tuberculosis, or other infectious diseases; no history of trauma or blood transfusion; and no known drug or food allergies. His personal history included 40 years of smoking (quit 5 years ago), with no alcohol abuse. His family history was unremarkable regarding bleeding disorders or genetic diseases. The patient underwent one percutaneous coronary PCI 3 months ago and received standard antiplatelet therapy including aspirin and clopidogrel (a P2Y12 inhibitor). No abnormal bleeding events occurred during or after the procedure. During this hospitalization, coronary angiography was performed without any abnormal bleeding events. He did not receive heparin, other anticoagulation therapy, FIB concentrate, or any replacement therapy, as the preoperative coagulation screening abnormalities were subsequently identified as assay interference due to FG-LM rather than true FIB deficiency, and the patient had no clinical evidence of bleeding risk.

It is noteworthy that the patient’s FIB level was measured at 3.76 g/L in a previous hospital (Hysocare CS-5100, Shanghai Sun Reagents) 1 week earlier. Repeat testing in our hospital over two consecutive days using the FIB optical method still showed <0.35 g/L, while the PT derivative method yielded <0.6 g/L (Table 1). The test results were inconsistent with the clinical manifestations, prompting the initiation of a laboratory re-examination protocol: ① retrieval of coagulation curves, ② switch to magnetic bead method, ③ normal plasma mixing test, ④ manual observation of clot morphology, and ⑤ submission for FGB gene sequencing.

**Table 1:** Patient-related test results.

Test	Day of hospitalization	The next day	The third day	Reference interval
PT (s)	11.8	11.8	11.2	9.4–12.5
APTT (s)	36.7	33.2	33.6	25.1–36.5
FIB (g/L)	<0.35	<0.35	<0.35	2.38–4.98
TT (s)	18.9	19.2	19.6	10.3–16.6
FIB-RP (g/L)	0.93	<0.6	<0.6	2.76–4.71
FDP (mg/L)	1.38	1.13	1.11	<2.01
D-D (mg/L)	0.125	0.128	0.111	0–0.243
AT (%)	75.0	69.0	64.0	83–128

PT: prothrombin time, APTT: activated partial thromboplastin time, FIB: fibrinogen Clauss method (reagent: Instrumentation Laboratory Company, USA); TT: thrombin time (reagent: Instrumentation Laboratory Company, USA); FIB-RP: fibrinogen PT derivative method; FDP: fibrinogen degradation product (reagent: Instrumentation Laboratory Company, USA); D-D: D-dimer (reagent: Instrumentation Laboratory Company, USA); AT: antithrombin III activity (reagent: Instrumentation Laboratory Company, USA).

### 2.1 Analysis and verification of clotting curve

**Figure 1:**
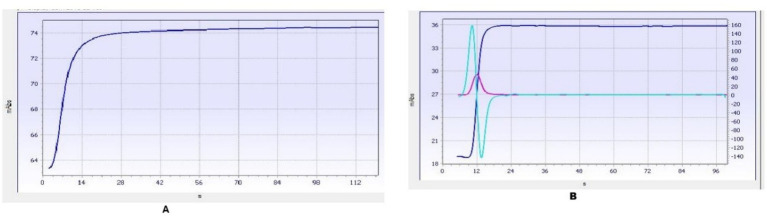
Clotting curve analysis of the patient. **(A)** Patient’s FIB clotting curve (Clauss method) shows fibrin formation but lacking a plateau phase, with ΔmAbs below the instrument threshold (15 mAU), resulting in a reported value of <0.35 g/L. The clotting time is paradoxically shortened. A normal control curve is shown for comparison, demonstrating a clear plateau phase and adequate amplitude. **(B)** Patient’s PT-derived FIB clotting curve showing morphological similarity to a low FIB control curve, with reduced amplitude and plateau. CT, clotting time; ΔmAbs, change in milli-absorbance units; mAU, milli-absorbance units; Th, threshold.

The ACL TOP-750 optical method calculates FIB by substituting the time corresponding to a 37% change in clotting absorbance threshold into the standard curve. The patient’s curve shows fibrin formation but lacks a plateau phase, with ΔmAbs below the instrument minimum requirement, thus reporting “<0.35 g/L.” Compared to the typical low FIB curve and normal FIB curve, the patient’s clotting time was paradoxically shortened, suggesting increased clot translucency rather than reduced FIB quantity. The patient’s PT-derived method curve exhibited morphological similarity to the low FIB control, indicating that end-point recognition errors in the optical method similarly affect the derived algorithm (Figure 1).

To validate the methodological change, the same specimen was retested using the STAGO Compact coagulation analyzer (magnetic bead method, DIAGNOSTICA STAGO, France) with STA-Fibrinogen 5 kit (DIAGNOSTICA STAGO, France). This system uses a mechanical (magnetic bead) detection principle: a steel bead is oscillated within the reaction cuvette, and as fibrin clot forms, the movement of the bead is progressively impeded. The endpoint is detected when the bead’s oscillation amplitude decreases below a defined threshold. This method is truly mechanical/magnetic, as it directly measures the viscoelastic properties of the forming clot rather than changes in turbidity or optical density. The FIB result was 4.18 g/L (reference range: 2–4 g/L), demonstrating methodological discrepancy compared to the optical method. Subsequently, the test was repeated using a thrombin reagent from Shandong Aikeda (China) on the ACL TOP-750 analyzer, with FIB-C still reporting <0.35 g/L (ΔmAbs below the instrument lower limit). This confirmed that the abnormal result was not related to the reagent brand but was determined by methodological principles (optical vs. magnetic bead) (2).

### 2.2 Normal plasma mixing test

After confirming the magnetic bead method, plasma from a patient with 4.18 g/L FG-LM was mixed with normal plasma (NPP, 3.10 g/L) in a gradient as shown in Table 2, and FIB was immediately measured using the ACL TOP-750 (optical method). When the patient plasma ratio was ≥1:1, ΔmAbs <15 mAU, and the instrument reported “FAILED”; readings were only obtainable at ratios ≤1:2. The second tube (1:2) measured 2.81 g/L, which was 18.8% lower than the theoretical value of 3.46 g/L, indicating that abnormal FIB reduced the overall clots’ translucency. As the ratio increased further, the signal completely disappeared, demonstrating dose-dependent optical signal interference (i.e., reduced turbidity signal due to the abnormal translucent clot). This finding is consistent with optical signal loss rather than the presence of an acquired inhibitor. This result directly confirmed that the FG-LM transparent clot interfered with the turbidimetric endpoint recognition, providing laboratory evidence for pseudo-low FIB. It is noteworthy that the patient’s FIB level was measured at 3.76 g/L 1 week prior to admission at another hospital using a SYSMEX CS-5100 analyzer (SYSMEX CORPORATION, Japan) with a FIB reagent (immunoturbidimetric method) manufactured by Shanghai Sun Biological Technology Co., Ltd. (China).

**Table 2:** Results of the linear mixing test between patient and normal plasma.

Number	Tube 1	Tube 2	Tube 3	Tube 4	Tube 5
Patient plasma (FIB 4.18 g/L)	50 μL	100 μL	150 μL	200 μL	250 μL
Normal plasma (FIB 3.10 g/L)	250 μL	200 μL	150 μL	100 μL	50 μL
Patient/normal plasma ratio	1:5	1:2	1:1	2:1	5:1
Theoretical value of mixed plasma FIB	3.28 g/L	3.46 g/L	3.64 g/L	3.82 g/L	4.00 g/L
Measured value of mixed plasma FIB	3.60 g/L	2.81 g/L	FAILED	FAILED	FAILED
Signal reduction ratio	Not applicable	18.8%	–	–	–

The “signal reduction ratio” (formerly termed “inhibition ratio”) refers to the percentage decrease in measured FIB value compared to the theoretical value in mixed plasma. This reduction is due to dose-dependent optical signal interference caused by the translucent clot formed by abnormal FIB, rather than functional antagonism or the presence of an acquired inhibitor.

### 2.3 Manual control trial

**Figure 2:**
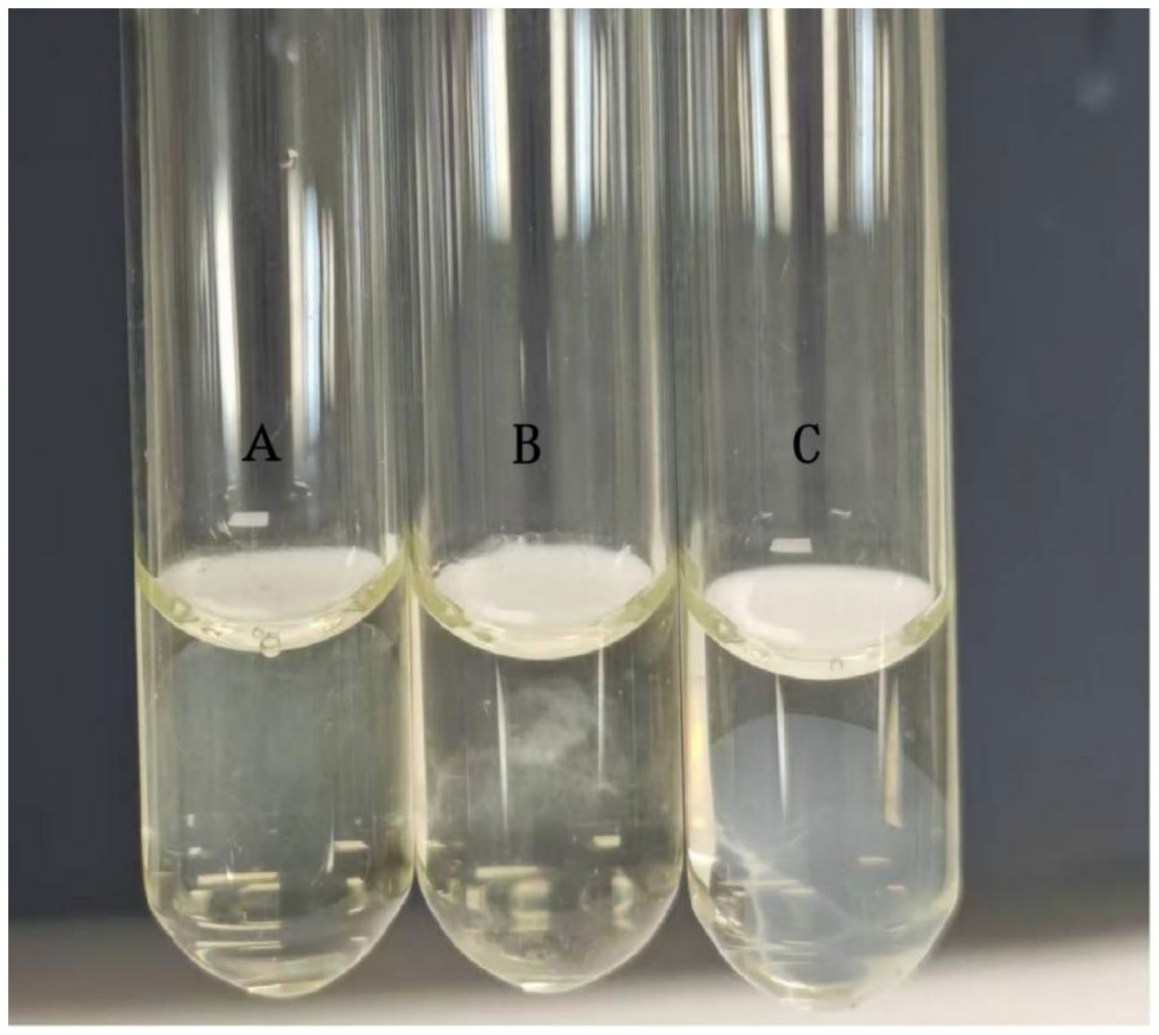
Manual Clauss method clot appearance. Three tubes are shown from left to right: **(A)** Low fibrinogen control plasma (FIB 1.08 g/L): Forms a less dense but still opaque clot with reduced volume. **(B)** Patient plasma (Fibrinogen Longmont, magnetic bead FIB 4.18 g/L): Forms a visibly transparent, loose, and fragile clot with poor mechanical strength, lacking the typical opaque appearance despite normal FIB concentration by mechanical method. This increased translucency reduces turbidity signal in optical Clauss assays, leading to falsely low or failed results. **(C)** Normal control plasma (FIB 4.63 g/L): Forms an opaque, firm, and well-contracted clot.

Based on the contradictory results of optical-magnetic bead assays and the dose-dependent inhibition observed in mixed experiments, the Clauss manual method was used for verification. A volume of 70 μL of patient plasma was mixed with 630 μL of factor diluent, pre-warmed at 37 °C, followed by the addition of 350 μL of thrombin reagent. The stopwatch was immediately started to observe clot formation. Results: The patient’s clotting time was significantly shorter than that of the normal control (4.63 g/L) and the low-FIB control (1.08 g/L). The clot was observed to be transparent, loose, and fragile (Figure 2). The transparent clot resulted in insufficient changes in absorbance, preventing the optical method from achieving the ΔmAbs > 15mAU threshold, thereby yielding a falsely low value. The manual method directly recorded the endpoint observed visually, unaffected by turbidity.

### 2.4 Genetic testing

Genetic testing ultimately confirmed the presence of a FGB gene c.586C > T heterozygous mutation, Sanger sequencing was performed on an ABI 3730XL DNA Genetic Analyzer (Thermo Fisher Scientific, Waltham, MA, USA), resulting in the substitution of arginine at position 196 with cysteine (p.Arg196Cys). This mutation is associated with a hereditary abnormal fibrinogenemia, known as FG-LM.

## 3 Discussion

The Bβ p.Arg196Cys mutation in FG-LM alters fibrin polymerization due to defective lateral association of protofibrils, resulting in increased clot translucency and pseudo-depletion or FAILED results in optical FIB assays. The magnetic bead method, which assesses end-point viscoelasticity, is generally less affected by increased clot translucency (1, 2). However, as highlighted by Jennings et al. (4) and Leung et al. (3), the laboratory findings in FG-LM are not simply dichotomous (optical suppressed vs. magnetic bead normal). Significant inter-laboratory and inter-platform variability exists, and normal Clauss FIB results may occasionally be obtained on optical analyzers depending on the specific thrombin reagent used. Thus, FG-LM may cause method-, reagent-, platform-, and algorithm-dependent discrepancies, with optical signal loss being a major but not exclusive mechanism. This case validates four key identification criteria: ① significantly lower or FAILED optical results with normal magnetic bead results, ② normal or shortened clotting time but ΔmAbs < 15 mAU, ③ FAILED optical results at plasma concentrations ≥1:1, demonstrating dose-dependent optical signal interference (reduced turbidity signal due to the abnormal translucent clot), ④ visible transparent and fragile clots on manual examination, providing direct evidence. These phenotypic-methodological differences offer a simple, rapid laboratory pathway for clinical identification of FG-LM (5), avoiding unnecessary blood product transfusions and surgical delays.

Since the first description by Lefkowitz et al. (1) in 2000, FG-LM has been reported in several studies. To provide a clear overview of dysfibrinogenemia, all published case studies are summarized in Table 3. Lefkowitz et al. (1) originally identified FG-LM in a family with prolonged clot-based test results and demonstrated that the BβArg166Cys mutation introduces a free thiol group, leading to impaired fibrin polymerization. The authors first noted that optical detection methods yielded falsely low FIB levels, while mechanical methods gave normal results. Lounes et al. (2) further characterized the molecular mechanism, showing that the impaired polymerization is not rescued by removing disulfide-linked dimers, and that the mutant FIB forms abnormal protofibrils with reduced lateral aggregation. Jennings et al. (4) conducted a multicenter study demonstrating significant inter-laboratory and inter-platform variability in FIB measurement for FG-LM samples, highlighting that results may vary substantially depending on the specific analyzer and reagent combination. Leung et al. (3) reported that normal Clauss FIB results may occasionally be obtained on optical analyzers depending on the thrombin reagent used, adding further complexity to the laboratory diagnosis of this condition. A summary of all reported FG-LM cases to date is presented in Table 3, including clinical presentations, laboratory findings, and management approaches.

**Table 3:** Summary of published fibrinogen Longmont cases.

First author	Year	Number of cases/families	Clinical presentation	Key laboratory findings
Lefkowitz et al. (1)	2000	One family (multiple members)	Prolonged bleeding time, easy bruising	Optical FIB ↓, mechanical FIB normal
Lounes et al. (2)	2001	Same family as Lefkowitz et al.	Asymptomatic or mild bleeding	Impaired fibrin polymerization
Jennings et al. (4)	2017	One sample (multicenter study)	Not specified	Marked inter-laboratory and inter-platform variability (normal on some analyzers, low on others)
Leung et al. (3)	2022	One case	Postpartum hemorrhage	Optical FIB normal with some reagents (e.g., STAGO), low with others, demonstrating reagent-dependent variability
Present case	2026	One case	Asymptomatic (unstable angina)	Optical FIB FAILED, magnetic bead normal

Clinical manifestations are highly heterogeneous. Both this case and literature reports include asymptomatic cases (2, 6); some families present with epistaxis or massive hemorrhage during delivery (2). The bleeding risk may be associated with trauma, hemostatic pressure during labor, or other coexisting coagulation defects. Preventive transfusion is not required for asymptomatic patients. However, the management of bleeding risk in patients with FG-LM or other dysfibrinogenemias during high-risk procedures requires careful consideration. It is important to distinguish between quantitative FIB disorders (e.g., afibrinogenemia or hypofibrinogenemia), where FIB replacement is clearly indicated, and qualitative disorders, such as FG-LM, where functional FIB levels may be normal despite abnormal polymerization. For patients with dysfibrinogenemia undergoing high-risk procedures (e.g., extracorporeal circulation and hepatectomy), Yan et al. recommended monitoring fibrinogen levels using mechanical or manual methods rather than optical assays. Based on their four-case series and literature review, they suggested a target functional fibrinogen level of >1.5–2.0 g/L. However, it should be noted that this target is extrapolated from quantitative FIB deficiency guidelines, and its applicability to dysfibrinogenemia is not firmly established by prospective studies. They also noted that asymptomatic patients may successfully undergo major surgery without prophylactic fibrinogen replacement (6). In asymptomatic patients like the one reported here, no prophylactic replacement was given, and the patient successfully underwent PCI without bleeding complications, supporting the view that routine prophylaxis may not be necessary in such cases. A comprehensive review of dysfibrinogenemia by Casini et al. (7) discussed the general clinical heterogeneity and management of various FIB variants. According to our literature search summarized in Table 3, the number of reported FG-LM cases to date remains limited, and the overall bleeding risk appears low. However, >15% of patients with dysfibrinogenemia in the review by Casini et al. required transfusion support during major surgeries or delivery, highlighting the need for individualized management.

The antithrombin activity findings: Table 1 shows persistently low AT activity values (75, 69, and 64%; reference range 83–128%) that were not discussed previously. Acquired AT deficiency due to heparin therapy, liver disease, or DIC was ruled out based on normal liver/kidney function, negative DIC markers, and no heparin exposure. Inherited AT deficiency is unlikely given the absence of personal/family thrombosis history. The progressive decline suggests a possible consumptive or hemodilutional effect related to the patient’s cardiovascular status. Importantly, dysfibrinogenemia does not directly cause AT deficiency, and no thrombotic events occurred in this patient. However, monitoring AT levels in similar cases may be prudent when additional risk factors are present. The synergistic effect of instruments and reagents can create a “window of normality.” Regarding reagent insert information on interferences, this study reviewed the package inserts of all FIB-related reagents, including the FIB (Clauss) kit (Instrumentation Laboratory Company, USA), the STA-Fibrinogen 5 kit (DIAGNOSTICA STAGO, France), and the immunoturbidimetric FIB reagent (Shanghai Sun Biological Technology Co., Ltd., China). None of these inserts provided specific information regarding analytical interferences caused by abnormal FIB molecules, including dysfibrinogenemia or variant FIB such as FG-LM. To further investigate this issue, the manufacturers of the ACL TOP-750 system (Instrumentation Laboratory Company) and the STAGO Compact system (DIAGNOSTICA STAGO) were contacted. Both manufacturers expressed that while the optical detection system is designed to measure FIB concentration based on turbidity changes, rare genetic variants affecting clot translucency may lead to falsely low or failed results. However, neither manufacturer provided specific validation data for dysfibrinogenemia interference in their current reagent inserts. This gap highlights the need for manufacturers to include warnings or validation information regarding hereditary dysfibrinogenemia in future reagent insert updates, as such information is crucial for laboratories encountering discrepant FIB results. This case validates the multicenter study by Jennings et al. (4): The same FG-LM plasma sample may yield normal values on Sysmex CS2100 but consistently low readings on ACL TOP or CA660. Conversely, Leung et al. (3) obtained elevated results with STAGO reagents on ACL TOP, indicating an interaction between thrombin concentration, detection wavelength, and mutant protein polymerization rate. A “normal” false appearance may occur when the ΔmAbs just exceed the instrument threshold. After laboratory reagents or instruments are replaced, retrospective review of previously suspected samples is recommended to avoid missed diagnoses.

Interpretation of mixed test boundaries: In this study, the 1:1 mixture yielded a FAILED result, with a complete loss of turbidity signal (100% signal reduction). The 1:2 mixture showed an 18.8% reduction in measured value compared to the theoretical value (2.81 g/L vs. 3.46 g/L). This reduction reflects dose-dependent optical signal interference due to the translucent clot formed by abnormal FIB, rather than functional antagonism or the presence of an acquired inhibitor. Therefore, the term “signal reduction” is preferred over “inhibition ratio” to avoid misinterpretation. Attention should be paid to “optical signal loss” and parallel comparison with the magnetic bead method to avoid incorrectly diagnosing an acquired inhibitor (8). FIB antigen testing was not performed in this case. Congenital dysfibrinogenemia is characterized by a discrepancy between functional and antigenic FIB. In the absence of direct antigen measurement, the PT-derived FIB (FIB-RP) value of 0.93 g/L serves as a useful surrogate, as PT-derived FIB has been shown to correlate reasonably well with antigenic FIB in dysfibrinogenemia (8). The normal PT-derived value (reference range 2.76–4.71 g/L) further supports that the patient had a qualitative defect (FG-LM) rather than quantitative FIB deficiency.

The four-step laboratory pathway (magnetic bead/hand-check → analysis of ΔmAbs → 1:1 pooled test → FGB sequencing) has been validated in multiple domestic centers (8), enabling rapid identification of FG-LM. Real-time communication between clinical and laboratory departments, along with multi-method validation, is crucial to avoid misdiagnosis.

As this is a single case report, the findings and diagnostic approach described herein may not be generalizable to all patients with FG-LM or other dysfibrinogenemia variants. The proposed four-step laboratory pathway should be considered a preliminary framework based on our experience with this case and a limited number of published reports. Multi-center studies with larger patient cohorts are needed to validate the diagnostic criteria and management recommendations discussed in this paper.

## Data Availability

The raw data supporting the conclusions of this article will be made available by the authors, without undue reservation.
